# Validity evidence for the Clinic Screening Tool for HIV (CST-HIV) addressing factors that undermine health-related quality of life in people living with HIV

**DOI:** 10.3389/fpsyg.2026.1940441

**Published:** 2026-10-16

**Authors:** María José Fuster-RuizdeApodaca, Patricia Recio, Trenton M. White, Carlos Iniesta, Rebeca Izquierdo, Julián Alexander Portocarrero, Jeffrey V. Lazarus, Inmaculada Jarrín

**Affiliations:** 1 Faculty of Psychology, Universidad Nacional de Educación a Distancia (UNED), Madrid, Spain; 2 Spanish Interdisciplinary AIDS Society (SEISIDA), Madrid, Spain; 3 Barcelona Institute for Global Health (ISGlobal), Barcelona, Spain; 4 City University of New York School of Public Health and Health Policy (CUNYSPH), New York, NY, United States; 5 CIBERINFEC, Carlos III Health Institute, Madrid, Spain; 6 CoRIS Cohort Coordination Unit, National Centre for Epidemiology, Carlos III Health Institute, Madrid, Spain

**Keywords:** HIV screening, long-term well-being, patient-reported outcomes, people aging with HIV, person-centered care

## Abstract

**Background:**

This study evaluated the psychometric properties and clinical validity of the Clinic Screening Tool for HIV (CST-HIV) to determine whether the CST-HIV demonstrates a reliable multidimensional structure and clinically meaningful associations with established patient-reported outcome measures (PROM) and HIV clinical markers.

**Methods:**

A cross-sectional study was conducted within the Spanish CoRIS multicentre cohort, including 286 adults with HIV. Participants completed the CST-HIV and validated PROMs. Clinical indicators (CD4^+^ cell count and percentage, HIV-1 RNA viral load, and non-AIDS events) were collected at current and retrospective time points. Analyses included confirmatory factor analysis, internal consistency, convergent and discriminant validity, associations with clinical markers, and age-group comparisons.

**Results:**

Confirmatory factor analysis showed good model fit, with satisfactory internal consistency across domains (ω = 0.86–0.89). CST-HIV shows convergent validity, including psychological distress with PHQ-4 (*r* = 0.79), anticipated stigma with HIV stigma (*r* = 0.65), sexuality with sexual satisfaction (*r* = −0.74), sleep and fatigue with insomnia severity (*r* = 0.79), and material deprivation with social exclusion (*r* = 0.67) (all *p* < 0.001). Anticipated stigma (ρ = −0.16, *p* = 0.014) and material deprivation (ρ = −0.18, *p* = 0.005) were associated with lower CD4^+^ counts, while material deprivation was associated with higher viral load (ρ = 0.13, *p* = 0.046). Sexual satisfaction was lower among participants aged ≥50 years (*d* = −0.47, *p* < 0.001).

**Conclusion:**

The CST-HIV demonstrates strong psychometric performance, supporting its use as a valid, brief, multidimensional screening tool to inform person-centered HIV care and clinical intervention.

## 1 Introduction

Advances in antiretroviral therapy (ART) have transformed HIV infection into a manageable chronic condition, providing people living with HIV (PLWH) the possibility of longer survival and improving their overall health status (Teeraananchai et al., 2017; Trickey et al., 2017). However, as clinical management has improved, attention has increasingly shifted toward a broader understanding of health and quality of life in PLWH, extending beyond viral suppression alone (Miners et al., 2014; Lazarus et al., 2016). Despite effective viral suppression, many PLWH continue to experience a wide range of physical, psychological, and social challenges as they age, including comorbid conditions, like atherosclerosis, metabolic dysfunction, kidney disease, Parkinson’s disease, depression, and anxiety, the latter two often derived from stigma and/or socioeconomic disadvantage (Asmelash and Nigatie, 2024; Degroote et al., 2014; Wilson et al., 2016; Chuah et al., 2017; Bristowe et al., 2020; Shorer et al., 2026; Paternò Raddusa et al., 2024; White et al., 2026).

Health-related quality of life (HRQoL) has become widely recognized as a crucial outcome in HIV care (Lazarus et al., 2016; World Health Organization, 2016; Guaraldi et al., 2019; World Health Organization (WHO), 2022). In clinical practice, systematic assessment of HRQoL and biopsychosocial domains is essential for identifying the most burdensome issues affecting PLWH, many of which go beyond what standard clinical and laboratory measures can detect (Fredericksen et al., 2020; Kall et al., 2020). In this context, patient-reported outcome measures (PROMs) have become increasingly valued as tools to capture the patient perspective and to support the delivery of person-centered care (Engler et al., 2017; Wheat et al., 2018).

While numerous validated PROMs exist in the field of HIV, many are lengthy or narrowly focused on single domains, such as medication adherence, symptoms, or stigma, making their use cumbersome in routine practice (Engler et al., 2017). The need for brief, multidimensional, and pragmatic screening tools suitable for routine implementation in specialist clinics has thus been emphasized in recent research and policy documents (Lazarus et al., 2016; Bristowe et al., 2020). To address this gap, our group previously developed and validated the Clinic Screening Tool for HIV (CST-HIV), a short PROM specifically designed to systematically identify eight core areas of concern for PLWH: anticipated stigma, emotional distress, sexuality, social support, material deprivation, sleep and fatigue, cognitive complaints, and physical symptoms (Fuster-Ruiz de Apodaca et al., 2021). Anticipated stigma refers to the subjective expectation or fear that one will experience prejudice, discrimination, stereotyping, or social rejection from others in the future due to their HIV-positive status. Emotional distress encompasses a broad range of negative psychological states and affective symptoms, including persistent anxiety, depressive mood, elevated stress, and emotional vulnerability. Sexuality addresses concerns related to intimate relationships, sexual function, changes in libido, fear of transmission, and the psychological impact of HIV status on sexual well-being and identity. Social support reflects the perceived availability, quality, and adequacy of emotional, informational, and practical resources provided by family, friends, partners, and community networks. Material deprivation describes the acute financial and structural hardships experienced by individuals, including economic instability, housing precariousness, food insecurity, and difficulty affording essential health or living expenses. Sleep and fatigue captures self-reported disruptions in sleep quality and architecture (e.g., insomnia, unrefreshing sleep) alongside chronic, debilitating physical and mental exhaustion that is unalleviated by rest. Cognitive complaints involves subjective difficulties in daily mental functioning, specifically self-perceived deficits in memory, concentration, processing speed, attention, and executive execution. Physical symptoms pertains to the burden, frequency, and severity of somatic manifestations and treatment side effects, including chronic pain, gastrointestinal disturbances, neuropathy, and physical weakness. The development of the CST-HIV followed best-practice guidelines for PROM development, involving literature review, qualitative research with multidisciplinary experts and PLWH, expert item selection and revision, cognitive debriefing, and extensive pilot testing (Eignor, 2001; Revicki et al., 2007; Fuster-Ruiz de Apodaca et al., 2021). In its initial psychometric evaluation, the CST-HIV demonstrated robust construct and criterion validity, supported by confirmatory factor analysis and strong correlations with standardized instruments assessing related domains, as well as good evidence for reliability and internal consistency (Fuster-Ruiz de Apodaca et al., 2021). Despite these promising results, further validation is necessary to assess the relationship between CST-HIV scores and clinical outcomes in broader, more representative cohorts.

Although previous studies have reported associations between psychosocial variables and clinical outcomes in PLWH, the evidence remains limited, especially for multidimensional PROMs suitable for rapid screening in everyday clinical care (Sok et al., 2018; Guaraldi et al., 2019). This study aims to reinforce and extend the evidence supporting the validity of interpretations derived from CST-HIV scores through a comprehensive evaluation of its psychometric properties in a large, multicenter cohort of PLWH in Spain. Specifically, the study examines evidence based on internal structure, and on relations with other variables, including patterns of associations that reflect theoretically expected convergence with related constructs. In addition, we evaluate evidence of criterion-related validity through associations with concurrent and retrospective clinical indicators, providing complementary information on the performance of CST-HIV scores in clinically relevant contexts.

## 2 Methods

### 2.1 Study design, participants, and data collection

We designed a cross-sectional study within the Spanish CoRIS cohort. CoRIS is an open, multicentre prospective cohort of HIV-positive adults, who were naïve to antiretroviral treatment at study entry, recruited since January 2004 from any of the 48 centers across 14 of the 17 autonomous regions in the Spanish public healthcare system (Sobrino-Vegas et al., 2011). A total of 17 CoRIS centers participated in this study, recruiting 286 PLWH among those who attended their outpatient clinic between June 2021 and June 2023.

HIV healthcare professionals informed eligible patients about the purpose of the study and invited them to take part. The general inclusion criteria were having an HIV-positive diagnosis, being at least 18 years old, and not having any severe psychiatric or cognitive disorders. Individuals who agreed to participate received a written informed consent form.

The procedures for collecting self-reported measures involved providing participants with access to a unique link to download a dedicated mobile application, which enabled them to complete the online questionnaire. Each participant received a personal login, allowing them to pause and resume the survey at their convenience. The application was developed specifically for this research, with an average completion time estimated at 14 min.

The CoRIS investigators obtained health variables and clinical markers from the cohort database. These data were collected at two distinct time points: a cross-sectional assessment concurrent with the administration of patient-reported outcomes (PROs) and a retrospective assessment corresponding to two years prior. CoRIS cohort was approved by the Clinical Research Ethics Committee of the Gregorio Marañón General University Hospital. All patients consented to join CoRIS and to participate in this study by signing both informed consent forms. This particular study received approval from the Clinical Research Ethics Committee Carlos III Health Institute, Madrid, Spain (CEI PI 94 _2018-v2).

### 2.2 Measures

Self-reported measures: The primary instrument administered in this study was CST-HIV, comprising 24 items reflecting core areas identified as relevant to well-being and quality of life among PLWH based on literature review, qualitative research, and expert consensus,(Fuster-Ruiz de Apodaca et al., 2021) and grouped into eight dimensions: (1) anticipated stigma, (2) emotional distress, (3) sexuality, (4) social support, (5) material deprivation, (6) sleep and fatigue, (7) cognitive complaints, and (8) physical symptoms. Each dimension is assessed through three targeted items, found in Supplementary information, enabling concise yet comprehensive screening. Items are answered using a five-point Likert scale, with higher summed scores indicating greater burden in each respective area.

To assess convergent validity, we included the following validated measures targeting similar constructs to the dimensions of the CST-HIV. For the dimension of anticipated stigma, we used the dimensions of disclosure concerns and public stigma from the Spanish Stigma Scale (Berger et al., 2001; Fuster-RuizdeApodaca et al., 2015).

For the dimension of emotional distress, we evaluated symptoms of depression and anxiety using the Patient Health Questionnaire-4 (PHQ-4) (Kroenke et al., 2009), a brief tool consisting of two items for anxiety and two for depression. For the dimension on sexuality, sexual satisfaction was measured via the PROMIS V2.0 Satisfaction with Sex Life (Weinfurt et al., 2015). To evaluate the dimension of social support, we used the Spanish version of the Duke-UNC Functional Social Support Questionnaire (Broadhead et al., 1988; Bellón et al., 1996).

For social exclusion, we used the Spanish adapted version of the Social Exclusion Index for Health Surveys (SEI-HS) (van Bergen et al., 2017). For the dimension of sleep and fatigue, we used the Spanish version of the Insomnia Severity Index (ISI) (Bastien, 2001; Fernandez-Mendoza et al., 2012) and the Spanish version of the seven-item Fatigue Severity Scale (FSS) (Fernández et al., 2011; Lerdal et al., 2011). For neurocognitive complaints, participants’ concerns about cognitive function were measured using the NeuroQoL V2.0 Cognitive Function scale (Lai et al., 2014). Two instruments evaluated quality of life: the disease-specific WHOQOL-HIV-BREF (Fuster-Ruizdeapodaca et al., 2019), validated for Spanish populations, and the EQ-5D-5L was used as a generic measure (Herdman et al., 2011).

The survey also captured sociodemographic characteristics, including age, duration of HIV infection, sex, gender, marital status, sexual orientation, country of origin, educational attainment, employment status, housing situation, household monthly income, and personal monthly income.

Health-related measures and objective clinical markers. These variables were obtained from the CoRIS database at two distinct time points: a cross-sectional assessment concurrent with the administration of patient-reported outcomes (PROs) and a retrospective assessment corresponding to 2 years prior. Diagnosis of an AIDS-defining illness, alcohol consumption measured as the number of standard alcoholic units consumed in the week preceding each assessment, current injecting drug use status at each time point, and tobacco consumption, including smoking status (current smoker—daily or occasional, former smoker, or non-smoker) and, for smokers, the daily number of cigarettes consumed, for both the current and retrospective periods. In addition, objective clinical markers were extracted from CORIS’s data base at both time points, including CD4^+^ cell count (cells/mm^3^) as an indicator of immune status, CD4^+^ cell percentage, viral load (copies/mL) reflecting virologic control, and the Non-AIDS Events Score (NAES), defined as the number of non-AIDS-defining events and used as an additional marker of morbidity. We used these objective clinical markers to assess the criterion validity of the CST-HIV instrument.

### 2.3 Statistical analysis

Continuous variables were summarized using means, standard deviations, and ranges, while categorical variables were described using frequencies and percentages.

Independent-samples t-tests were employed to compare CST-HIV domain scores across demographic groups, including age category (<50 vs. ≥50 years), gender, history of AIDS diagnosis, and sexual orientation (heterosexual vs. men who have sex with men, MSM). Where statistically significant differences were identified, mean differences and effect sizes (Cohen’s d) were reported.

A confirmatory factor analysis (CFA) was conducted using the WLSMV estimator on polychoric correlations to test the hypothesized eight-factor structure of the CST-HIV instrument. Model fit was evaluated using multiple indices: chi-square to degrees of freedom ratio (χ^2^/df), Comparative Fit Index (CFI), Tucker-Lewis Index (TLI), Bentler-Bonett Non-Normed Fit Index (NNFI), Normed Fit Index (NFI), Incremental Fit Index (IFI), Relative Noncentrality Index (RNI), Goodness of Fit Index (GFI), Root Mean Square Error of Approximation (RMSEA) with 90% confidence interval, and Standardized Root Mean Square Residual (SRMR). Sampling adequacy was tested with the Kaiser-Meyer-Olkin measure, and assumptions were verified with Bartlett’s test of sphericity. Composite reliability (ω) and Cronbach’s alpha (α) were also reported for each domain.

Convergent validity was assessed using Pearson’s correlation coefficients to examine associations between the eight CST-HIV dimensions and established instruments measuring theoretically related constructs.

Spearman’s rank correlations were computed between CST-HIV domain scores and clinical markers (CD4^+^ cell count, CD4^+^ cell percentage, viral load) at both time points (cross-sectional and retrospective). For viral load, correlations were conducted on the log10 scale; undetectable was defined as <50 copies/mL and values below the limit of detection were imputed as LOD/2 for log10 analyses. This approach is commonly used when handling left-censored biomarker data, including HIV viral load measurements (Hughes, 2000). Additionally, a prespecified sensitivity analysis used Pearson correlations with raw copies/mL for the retrospective time point to mirror legacy reporting.

To examine the associations of CST-HIV scores with changes in clinical markers over time, repeated measures modeling was performed. CD4^+^ count and CD4^+^ percentage were analyzed with a repeated-measures GLM, whereas viral load (log10) was analyzed with a marginal GEE to obtain population-averaged effects with robust standard errors.

Each model included Time (retrospective vs. cross-sectional), age group (<50 vs. ≥50), and all CST-HIV domains. The sample comprised 177 participants (116 under 50 years old, 61 over 50 years old). Multivariate and univariate tests were applied to identify significant associations. Parameter estimates were examined to clarify the direction of these associations. Complementary paired within-subject comparisons (paired t-test or Wilcoxon, as appropriate) were used to summarize change in continuous clinical markers, and McNemar tests were used for paired proportions (e.g., undetectable <50 copies/mL).

All statistical tests were two-tailed, with αset at 0.05. Effect sizes (Cohen’s d, partial eta squared) are reported to aid the interpretation of group differences and model effects. Correlation analyses were based on all available observations, whereas CFA, regression, and repeated-measures models were estimated using complete cases. Missingness mechanisms were not formally evaluated. Missing data for the main clinical indicators ranged from 18.2 to 21.7%. All data analysis was conducted using SPSS version 30 and JASP version 0.95.4.

## 3 Results

### 3.1 Participants’ characteristics

A total of 286 PLWH participated in the study, and their sociodemographic characteristics are shown in Table 1. The mean age was 43.9 ± 10.60. The majority self-identified as men and homosexual, and most reported being either single or married/cohabiting. The sample was predominantly comprised of participants residing in Spain, with a smaller proportion from other regions, including Latin America and Western Europe. Educational attainment within the sample was generally high, with most having completed secondary or higher education. In terms of employment, the majority were active in the workforce, while smaller groups were retired, unemployed, or students (Table 1).

**Table 1:** Participants’ sociodemographic characteristics.

Characteristic	Sub-characteristic	*n* = 286
Gender	Male	259 (90.6%)
	Female	25 (8.7%)
	Other	2 (0.7%)
Sexual orientation	Heterosexual	38 (13.3%)
	Homosexual	209 (73.1%)
	Bisexual	27 (9.4%)
	Other	4 (1.4%)
	Prefer not to answer	8 (2.8%)
Age (years)		43.9 ± 10.6
Marital status	Married or in a relationship	127 (44%)
	Divorced or separated	24 (8.4%)
	Single	129 (45.1%)
	Widowed	6 (2.1%)
Region	Spain	195 (68.7%)
	Western Europe	10 (3.5%)
	Eastern Europe	4 (1.4%)
	Sub-Saharan Africa	1 (0.4%)
	Latin America	70 (24.6%)
	Other	3 (1.1%)
	Unknown	1 (0.4%)
Educational level	No formal education	2 (0.7%)
	Primary education	25 (8.7%)
	Secondary education	99 (34.6%)
	Higher education	155 (54.2%)
	Other	5 (1.7%)
Employment status	Active worker	225 (78.7%)
	Retired or pensioner	14 (4.9%)
	Unemployed	28 (9.8%)
	Student	9 (3.1%)
	Other	10 (3.5%)
Housing situation	Own home	226 (79%)
	Living with family	26 (9.1%)
	Shared apartment	26 (9.1%)
	Living in someone else’s home	5 (1.7%)
	Institutional-provided housing	1 (0.3%)
	Other	2 (0.7%)
Household income (net €) per month	No income	4 (1.4%)
	≤300	6 (2.1%)
	301–600	10 (3.5%)
	601–900	14 (4.9%)
	901–1,200	39 (13.6%)
	1,201–1,800	48 (16.8%)
	1,801–2,400	47 (16.4%)
	2,401–3,000	41 (14.3%)
	3,001–4,500	38 (13.3%)
	4,501–6,000	20 (7.0%)
	Prefer not to answer	19 (6.6%)
Personal income	No income	13 (4.5%)
	≤300	17 (5.9%)
	301–600	18 (6.3%)
	601–900	23 (8.0%)
	901–1,200	40 (14%)
	1,201–1,800	68 (23.8%)
	1,801–2,400	44 (15.4%)
	2,401–3,000	25 (8.7%)
	3,001–4,500	14 (4.9%)
	4,501–6,000	7 (2.4%)
	Prefer not to answer	17 (5.9%)
Transmission route	Sexual intercourse	242 (84.6%)
	Sharing injection material	4 (1.4%)
	Transfusion exposure	2 (0.7%)
	Not known	36 (12.6%)
	Other	2 (0.7%)

Data are presented as *n*(%) or mean ± standard deviation.

### 3.2 Health-related data

In the cross-sectional assessment, most participants (98.9%) reported no prior AIDS diagnosis. In contrast, retrospective data revealed a somewhat higher proportion (11.6%) of AIDS diagnoses. Regarding alcohol consumption, nearly 40% of the sample provided data, of which more than half (56.1%) reported no alcohol use in the preceding week. The remainder showed a broad distribution of consumption, with 14.9% reporting consumption of a single unit, 14% reporting between 2 and 4 units, and a tiny percentage (<2% for each category) reporting heavier consumption, up to 40 units per week. In the retrospective period, among nearly 70% of participants providing data, the majority (59.5%) again reported abstention from alcohol, with similar proportions distributed across consumption categories and isolated cases reporting up to 42 units per week. No participants reported current injecting drug use at the time of the cross-sectional survey, and only one participant (0.5%) reported such use retrospectively. In the cross-sectional data, (*n* = 112), 36.6% reported daily smoking, 1.8% occasional smoking, and 19.6% former smoking. Daily cigarette consumption ranged widely, most frequently 10 or 20 cigarettes per day. Retrospective responses reflected a similar pattern: 29% identified as daily smokers, 4.1% as occasional smokers, 22.6% as ex-smokers, and 43.8% as non-smokers (see Supplementary Table S1).

### 3.3 Clinical markers

Table 2 shows participants’ clinical markers assessed at two time points: the cross-sectional time (Tc) and the retrospective time (Tr). CD4^+^ cell count did not differ between time points (median [IQR] Tr: 820 [580–1,034] vs. Tc: 764 [570–1,045]; Wilcoxon *p* ≥ 0.05; paired t-test *p* ≥ 0.05). By contrast, CD4^+^ percentage increased from Tr to Tc (mean difference = +1.55; 95% CI 0.70–2.40; Wilcoxon *p* < 0.001; paired t-test *p* < 0.001). For viral load, the proportion undetectable (<50 copies/mL) was similar at both points (Tc 94.1% vs. Tr 91.4%; McNemar *p* ≥ 0.05), while the quantitative level (log10) was slightly lower at Tc (mean difference = −0.127 log10; 95% CI − 0.241 to −0.013; *p* < 0.05). Finally, non–AIDS-related events (NAES) were less frequent at Tc than at Tr (mean difference = −0.189; 95% CI − 0.262 to −0.116; paired t-test *p* < 0.001; Wilcoxon *p* < 0.001), and the proportion with ≥1 event decreased from 16.78% at Tr to 4.20% at Tc (McNemar *p* < 0.001).

**Table 2:** Clinical outcomes at two time points (Tc and Tr).

Variable	Paired *N*	Tc	Tr	Change (Tc − Tr)	Test	*p*
CD4^+^ count (cells/mm^3^)	177	764 [570–1,045]^a^	820 [580–1,034]^a^	Median diff: 22 [−129–132]	Wilcoxon	0.649
				(Mean diff: 1.40; 95% CI − 36.1 to 38.9)	Paired *t*-test	0.942
CD4^+^ percentage (%)	174	34.57 ± 9.60^b^	33.02 ± 9.53^b^	Mean diff: 1.55 (95% CI 0.70–2.40)	Paired *t*-test	<0.001
HIV-1 RNA, log10 (copies/mL)	185	1.45 ± 0.30^c^	1.58 ± 0.74^c^	Mean diff: −0.127 (95% CI − 0.241 to −0.013)	Paired *t*-test	0.029
Geometric mean (copies/mL)	185	28.1	37.6	GM ratio (Tc/Tr): 0.747 (95% CI 0.574–0.971)	—	—
Undetectable <50 copies/mL, *n*/*N* (%)	185	174/185 (94.1%)^d^	169/185 (91.4%)^d^	Δ = +2.7 percentage points	McNemar	0.383
NAES (count per participant)	286	0.042 ± 0.20^b^	0.231 ± 0.61^b^	Mean diff: −0.189 (95% CI − 0.262 to −0.116)	Paired *t*-test	<0.001
					Wilcoxon	<0.001
≥1 NAES, n/N (%)	286	12/286 (4.20%)^d^	48/286 (16.78%)^d^	—	McNemar	<0.001

Difference defined as Tc − Tr (positive = increase from Tr to Tc). Paired data used in all analyses. Legend of superscripts: ^a^Median [IQR]; ^b^Mean ± SD; ^c^log10, Mean ± SD; ^d^*n*/*N* (%). Viral load reporting: log10 scale; undetectable defined as <50 copies/mL. Undetectable values were imputed as 25 copies/mL (LOD/2) for log10 analyses.

### 3.4 Descriptives of the patient reported measures (PROS)

Table 3 shows the descriptive statistics of the PROs used in the study. Participants reported moderate levels of internalized stigma. Depressive and anxiety symptoms were most frequently reported at mild to moderate levels. According to the scale’s cut-off points, 21.7% of participants exceeded the thresholds for probable clinical depression and 19.2% for probable clinical anxiety. Sexual satisfaction was generally favorable. Both confidant and affective social support, measured with the Duke-UNC scales, were robust across the sample, with most participants reporting substantial support in their close relationships. Insomnia symptoms, assessed with the Insomnia Severity Index, and fatigue, assessed with the Fatigue Severity Scale, were present to a moderate extent across the group. According to the established cutoffs for these scales, some 50.7% of the sample met criteria for severe insomnia and 35.7% for moderate insomnia. A total of 40.7% met criteria for severe fatigue. Neurocognitive complaints clustered toward the lower half of the possible range, suggesting that most participants did not experience severe difficulties in this area. Most participants (55.6%) were categorized as having mild cognitive impairment, while 22.7% were considered to have normal cognitive functioning. Moderate and severe cognitive impairment was observed in 18.9 and 2.8% of the sample, respectively. Classification of total social exclusion scores showed that 40.2% of participants were categorized as having low exclusion, 40.9% as moderate, and 18.9% as high/severe exclusion. Within the specific subdomains, limited social participation was low for 35.0% of participants, moderate for 40.9%, and high/severe for 24.1%. Material deprivation showed a more favorable profile, with the majority (56.3%) classified as low, 29.7% as moderate, and 14.0% as high/severe deprivation. In contrast, lack of normative integration was more pronounced, with 43.4% of participants experiencing high or severe disintegration. Regarding HRQoL, participants reported means between 14 and 15 in general, physical, and psychological health, as well as independence, social relationships, and environmental health. The dimension of SRPB presented the lowest mean. Scores tended to cluster in the upper range, reflecting a relatively preserved quality of life for most of the sample. The overall profile of this sample indicates that, although some difficulties and vulnerabilities were noted, most of the cohort reported functioning and well-being levels within favorable or moderate ranges.

**Table 3:** Descriptive statistics for patient-reported outcome measures (PROs).

Variable (instrument)	Dimension	M	SD	Skewness	Kurtosis	Min	Max
Stigma (Berger et al.)	Internalized stigma	2.84	0.66	−0.14	−0.60	1.12	4.00
Emotional Distress (PHQ-4)	Depression	1.61	1.53	0.94	0.49	0.00	6.00
	Anxiety	1.67	1.65	1.02	0.68	0.00	6.00
Sexuality (PROMIS v2.0)	Sexual satisfaction	3.27	0.94	−0.18	−0.52	1.00	5.00
Social Support (Duke-UNC)	Confidant support	3.79	1.18	−0.83	−0.35	1.00	5.00
	Affective support	3.72	1.14	−0.80	−0.36	1.00	5.00
Insomnia (ISI)	Insomnia severity	8.00	5.59	0.35	−0.75	0.00	23.00
Fatigue (FSS)	Fatigue severity	3.33	1.40	0.41	−0.77	1.00	6.44
Neurocognitive (PROMIS)	Cognitive complaints	27.16	4.22	−1.18	1.54	12.00	33.00
Social Exclusion (Adapted)	Lack of social participation	8.22	2.84	0.64	−0.56	5.00	15.00
	Material deprivation	6.89	1.93	0.91	−0.25	5.00	12.00
	Lack of normative integration	4.37	1.03	0.29	−0.76	3.00	7.00
	Total exclusion	19.48	4.39	0.63	−0.19	13.00	33.00
HRQoL (WHOQOL-HIV-BREF)	General health	14.13	3.31	−0.29	−0.35	4.00	20.00
	Physical health	15.01	3.02	−0.47	0.01	4.00	20.00
	Psychological health	14.48	2.95	−0.45	−0.22	6.40	20.00
	Independence	15.48	2.99	−0.44	−0.18	5.00	20.00
	Social relationships	14.55	3.18	−0.29	−0.39	6.00	20.00
	Environmental health	15.25	2.55	−0.34	−0.01	7.50	20.00
	SRPB	13.76	3.59	−0.16	−0.78	5.00	20.00

Scale ranges: Stigma Scale (1–4); PHQ-4 (0–3); PROMIS v2.0 Sexual Satisfaction (1–5); Duke-UNC Social Support (1–5); ISI (0–4); 6 FSS (1–7); 7 PROMIS Cognitive Function (1–5); 8 Social Exclusion (1–3); 9 WHOQOL-HIV-BREF (1–5).

Additional analyses, including confirmatory factor analysis, convergent and discriminant validity (Supplementary Table S2), criterion validity, and the Pearson correlation matrix of study variables (Table 3) are presented in Supplementary information. Confirmatory factor analysis supported the hypothesized eight-factor structure of CST-HIV, with moderate-to-high factor loadings across all domains (Table 4). The model showed a good fit to the data (χ^2^/df = 1.79, CFI = 0.972, TLI = 0.965, NNFI = 0.965, RMSEA = 0.053, and SRMR = 0.054).

**Table 4:** Standardized factor loadings and 95% confidence intervals for the CST-HIV eight-factor model.

Factor	Item	λ	LL 95% CI	UL 95% CI
F1. Anticipated stigma	Item 1	0.72	0.66	0.79
	Item 2	0.96	0.92	1.00
	Item 3	0.94	0.90	0.98
F2. Psychological distress	Item 4	0.88	0.84	0.92
	Item 5	0.92	0.88	0.95
	Item 6	0.76	0.70	0.82
F3. Sexuality	Item 7	0.49	0.36	0.62
	Item 8	0.76	0.67	0.85
	Item 9	0.81	0.72	0.90
F4. Social support	Item 10	0.82	0.72	0.92
	Item 11	0.91	0.81	1.00
	Item 12	0.80	0.71	0.90
F5. Material deprivation	Item 13	0.61	0.51	0.72
	Item 14	0.68	0.58	0.79
	Item 15	0.70	0.59	0.80
F6. Sleep and fatigue	Item 16	0.72	0.65	0.79
	Item 17	0.63	0.54	0.72
	Item 18	0.90	0.84	0.96
F7. Cognitive complaints	Item 19	0.92	0.88	0.96
	Item 20	0.93	0.90	0.97
	Item 21	0.81	0.75	0.86
F8. Physical symptoms	Item 22	0.64	0.54	0.73
	Item 23	0.73	0.64	0.81
	Item 24	0.73	0.65	0.81

λ = standardized factor loading; LL = lower limit; UL = upper limit. All factor loadings were statistically significant (*p* < 0.001).

## 4 Discussion

This study extends and reinforces the validity evidence for the CST-HIV by demonstrating a robust internal structure, strong convergent validity, and clinically meaningful associations with immunovirological markers in a multicentre cohort of people living with HIV (PLWH) in Spain (Wilson et al., 2016; Fuster-Ruiz de Apodaca et al., 2021). The results confirm that CST-HIV operates effectively as a brief, multidimensional patient-reported outcome measure (PROM) that efficiently identifies biopsychosocial burdens undermining health-related quality of life (HRQoL) in routine HIV care (Lazarus et al., 2016; Guaraldi et al., 2019).

Our findings replicate and extend the psychometric evidence from the initial development study, which reported the same structure and satisfactory internal consistency (Fuster-Ruiz de Apodaca et al., 2021). Collectively, the results indicate that CST-HIV captures distinct yet interrelated biopsychosocial dimensions known to shape HRQoL among PLWH. These dimensions are consistently identified in the literature as key determinants of HRQoL among PLWH (Degroote et al., 2014; Bristowe et al., 2020). Reliability indices demonstrate that CST-HIV scores are stable and internally consistent, even within a heterogeneous, real-world clinical cohort. Many existing HIV PROMs are either lengthy or focused on single domains, which limits their feasibility in busy clinical settings (Engler et al., 2017; Bristowe et al., 2020). In contrast, CST-HIV achieves an appropriate balance between brevity and psychometric robustness, aligning with recent recommendations that emphasize brief, pragmatic tools for integration into specialist HIV services (Lazarus et al., 2016; Kall et al., 2020).

The observed pattern of correlations with validated instruments provides strong evidence for the convergent and discriminant validity of CST-HIV. Each dimension demonstrated its strongest associations with conceptually related constructs, while correlations with more distal constructs, such as generic HRQoL, were weaker but in the expected direction. This pattern aligns with evidence that multidomain PROMs capture related but non-redundant aspects of patients’ experiences (Fuster-Ruizdeapodaca et al., 2019; Bristowe et al., 2020). Furthermore, CST-HIV dimensions were negatively associated with both disease-specific and generic HRQoL indicators, indicating that higher burden consistently corresponds to poorer perceived quality of life, consistent with previous research demonstrating that symptoms, stigma, and psychosocial stressors remain central determinants of HRQoL, even in the context of virological suppression (Lazarus et al., 2016; Guaraldi et al., 2019).

Cross-sectionally, higher material deprivation and anticipated stigma were associated with lower CD4^+^ cell counts. These results are consistent with prior research connecting unmet basic needs and socioeconomic vulnerability to poorer health outcomes and HRQoL among PLHIV (Degroote et al., 2014; Sok et al., 2018). Psychological distress showed a small between-subjects association with overall CD4^+^ count across the concurrent and retrospective assessments (partial η^2^ = 0.023, *p* = 0.048), with a positive coefficient at the retrospective assessment. The Time × Psychological distress interaction was not statistically significant; thus, no association with within-person change in CD4^+^ count was detected. Although collinearity diagnostics indicated no problematic multicollinearity among CST-HIV domains (VIFs = 1.12–2.05; tolerances = 0.49–0.90), these diagnostics do not explain the direction of the association or exclude residual confounding. Differences in ART duration, treatment and disease history, healthcare engagement, and psychosocial factors not accounted for in these models may have contributed to this finding. Selection processes related to survival, continued attendance in care, and availability of complete data also cannot be excluded. Because psychological distress was assessed only once, the analysis cannot establish temporal direction or distinguish reverse causation from differences in symptom reporting or shared determinants. Previous studies have reported partially comparable unexpected associations, including higher odds of generalized anxiety disorder with higher nadir CD4^+^ counts, or increasing CD4^+^counts without parallel changes in anxiety or depression (Santana et al., 2019; Shi et al., 2020). A separate CoRIS study of long-term survivors of advanced HIV disease found that a higher baseline CD4^+^/CD8 ratio was associated with lower subsequent scores in the spirituality, religion and personal beliefs domain of HRQoL; however, the different biomarker, outcome and selected population preclude interpreting this as replication of the present finding (Portilla-Tamarit et al., 2025). These observations provide context, not evidence of a direct biological relationship. Our finding should therefore be considered hypothesis-generating and requires independent longitudinal replication.

GLM results also showed that anticipated stigma was the only domain significantly associated with viral load, both as a small between-subjects effect and though a significant interaction with time. It indicates that participants with higher anticipated stigma had higher viral load at the retrospective time with attenuation of this difference at the cross-sectional time, suggesting that the detrimental impact of expecting future discrimination on virological control may weaken over time in individuals who remain engaged in care. This pattern is consistent with cross-sectional and longitudinal evidence showing that HIV-related stigma exerts a modest but robust influence on viremia, largely via depressive symptoms, ART adherence and medical mistrust, rather than through general symptom burden alone (Katz et al., 2013; Christopoulos et al., 2019, 2020; Kemp et al., 2019; Yigit et al., 2025). The magnitude and direction of the adjusted anticipated stigma effect in our sample remain in line with the CST-HIV conceptualization of anticipated stigma as specific social mechanism – rooted in disclosure concerns and expectations of public stigma- that is closely tied to HIV care cascade and virological outcomes, over and above other burden domains as emotional distress, fatigue or material deprivation (Fuster-Ruiz de Apodaca et al., 2021; Safreed-Harmon et al., 2022).

In group comparisons, CST-HIV identified significant age-related differences only in the sexuality domain, with younger participants reporting greater sexual satisfaction. No age differences were observed for stigma, psychological distress, social support, social exclusion, sleep and fatigue, cognitive complaints, or physical symptoms. This pattern indicates that sexuality remains a particularly sensitive and affected domain among older PLWH, warranting targeted assessment and intervention, while other psychosocial burdens are prevalent across age groups (Bristowe et al., 2019). The lack of consistent differences by gender or AIDS history, and the limited differences by sexual orientation, suggest that CST-HIV captures a broadly shared burden profile in this cohort, with specific nuances that can inform more tailored responses. From a person-centered care perspective, these findings support the use of CST-HIV to identify both common and subgroup-specific priorities that might otherwise be overlooked in time-pressured consultations (Fredericksen et al., 2020; Kall et al., 2020).

In conjunction with previous research, the current findings position CST-HIV as a promising tool for integrating systematic patient-reported assessment into routine HIV care. Implementation studies in HIV have demonstrated that routine use of PROMs can improve detection of symptoms and risk behaviors, enhance clinician–patient communication, and support shared decision-making, provided that clear follow-up pathways are established (Fredericksen et al., 2020; Kall et al., 2020). The brevity of CST-HIV (24 items) and its coverage of eight domains that align with internationally recognized HRQoL priorities make it particularly suitable for digital or waiting-room completion, followed by focused discussion of flagged areas during the consultation (Fuster-Ruiz de Apodaca et al., 2021).

A long-term approach using the CST-HIV might enable the assessment of its associations with future clinical events and HRQoL states. Such data are essential to support the implementation of the CST-HIV as a screening tool that not only identifies current priority needs but also informs intervention planning in real-world clinical workflows (Fredericksen et al., 2020; Kall et al., 2020). However, effective implementation will require attention to professional training, integration into electronic health records, and the availability of psychological, social, and economic support resources to address identified problems (Fredericksen et al., 2020). Methodologically rigorous implementation research is needed to explore how routine CST-HIV use influences clinical practice, HRQoL, and long-term health outcomes across diverse care settings and populations.

The major strengths of this study include the use of a multicentre clinical cohort, integration of PROMs with detailed clinical data at two time points, and a comprehensive psychometric evaluation encompassing internal structure, convergent and discriminant validity, and criterion-related evidence. However, several limitations should be acknowledged. The design remains largely cross-sectional with short-term retrospective clinical data, which limits statistical causal inference. The sample, although geographically diverse within Spain, may not represent all PLWH in other cultural or healthcare contexts. In particular, the sample was predominantly composed of men and included a relatively high proportion of participants with secondary or higher educational attainment. These characteristics are consistent with the profile of individuals engaged in regular HIV care within the participating centers but may limit the generalizability of the findings to women, individuals with lower educational levels, and other populations that may experience different psychosocial, economic, and healthcare-related challenges. Future studies should examine the psychometric performance and clinical utility of the CST-HIV in more socio-demographically diverse samples to strengthen the external validity of the instrument. Additionally, some clinical and behavioral variables had missing data or relied on self-report, potentially introducing measurement bias (Degroote et al., 2014; Sok et al., 2018). Missingness mechanisms were not formally assessed, and complete-case analyses may have introduced some degree of bias. Moreover, there are multiple factors that may influence the health of people living with HIV that are not captured in cohort studies, thereby limiting the ability to identify associations between the variables under study and clinical markers, as well as to elucidate the mechanisms operating through the associations observed.

In summary, this study validates the CST-HIV as a practical instrument for both clinical practice and research, and supports its integration as a core component of person-centered HIV care. Systematic and multidimensional assessment of patient-reported burdens should be regarded as a fundamental pillar alongside virological and immunological control (Lazarus et al., 2016; Guaraldi et al., 2019). Therefore, embedding the CST-HIV into routine consultations equips providers with a rapid, structured tool to identify multidimensional burdens and streamline targeted referrals to mental health, social, or palliative services. Future research should leverage the CST-HIV as a longitudinal endpoint to evaluate person-centered interventions, establish diagnostic cut-off thresholds, and investigate its utility for care retention and ART adherence.

## Data Availability

The raw data supporting the conclusions of this article will be made available by the authors, without undue reservation.

## Group members of CoRIS Cohort

Group authorship: Centers and investigators involved in CoRIS cohort are given below: Santiago Moreno, Inma Jarrín, David Dalmau, M Luisa Navarro, Federico Garcia, Eva Poveda, Jose Antonio Iribarren, Félix Gutiérrez, Francesc Vidal, Juan Berenguer, Juan González, CoRIS Executive Committee; Centers and investigators involved in CoRIS cohort are listed below: Inma Jarrín, Cristina Moreno, Marta Rava, Rebeca Izquierdo, Cristina Marco-Sánchez, Teresa Gómez-García, CoRIS Coordination Unit; José Luis Jiménez, BioBanK HIV Hospital General Universitario Gregorio Marañón; Sergio Reus, Irene Portilla, Esperanza Merino, Gema García, José Sánchez-Payá, Juan Carlos Rodríguez, Livia Giner, Joaquín Portilla, Vicente Boix, Diego Torrus, Julia Portilla-Tamarit, Héctor Pinargote, Hospital General Universitario Dr Balmis (Alicante); María Remedios Alemán, Nereyda Tosco-García, Ana López Lirola, Dácil García, Felicitas Díaz-Flores, María del Mar Alonso, Ricardo Pelazas, María Inmaculada Hernández, Lucia Romero-Acevedo, Abraham Bethencourt-Padilla, Daniel Rodríguez-Díaz, Ana María Godoy-Reyes, Hospital Universitario de Canarias (San Cristóbal de la Laguna); Víctor Asensi, Rebeca Cabo-Magadan, Lorena Fernández, Javier Díaz-Arias, Hospital Universitario Central de Asturias (Oviedo); Federico Pulido, Rafael Rubio, M Asunción Hernando, Otilia Bisbal, David Rial-Crestelo, María de Lagarde, Laura Bermejo, Mireia Santacreu, Juan Martín Torres, Belén Sánchez-López, Hospital Universitario 12 de Octubre (Madrid); José Antonio Iribarren, Xabier Kortajarena, Claudia Nevado Pavón, Xabier Camino, Miguel Ángel Goenaga, M Jesús Bustinduy, Harkaitz Azkune, Maialen Ibarguren, Ignacio Álvarez-Rodriguez, Leire Gil-Alonso, Francisco Carmona-Torre, Ana Bayona Carlos, Maialen Lekuona Sanz, Leire Balerdi Sarasola, Ander Goyache Moreno, Servicio de Enfermedades Infecciosas. Hospital Universitario Donostia, Instituto de Investigación Sanitaria BioGipuzkoa (Donostia-San Sebastián); Félix Gutiérrez, Catalina Robledano, Mar Masiá, Sergio Padilla, Rafael Pascual, Marta Fernández, Antonio Galiana, José Alberto García, Xavier Barber, Javier García Abellán, Guillermo Telenti, Ángela Botella, Paula Mascarell, Lidia García-Sánchez, Nuria Ena, Leandro López, Jennifer Vallejo, Nieves Gonzalo-Jiménez, Montserrat Ruiz, Christian Ledesma, Santiago López, María Espinosa-Pérez, Ana Quiles, María del Mar Alcalde-Encinas, José García-García, Rosario Hernández-Ros, José Carlos Escribano, Marouane Menchi-Elanci, María del Mar García Navarro, Melissa Bello Pérez, Hospital General Universitario De Elche (Elche); Roberto Muga, Daniel Fuster, Hospital Universitari Germans Trias i Pujol (Can Ruti) (Badalona); Cristina Diez, Isabel Gutiérrez, Juan Berenguer, Margarita Ramírez, Teresa Aldamiz-Echevarría, Francisco Tejerina, Leire Pérez-Latorre, Chiara Fanciulli, Saray Corral, Hospital General Universitario Gregorio Marañón (Madrid); Joaquim Peraire, Anna Rull, Anna Martí, Consuelo Viladés, Beatriz Villar, Lluïsa Guillem, Silvia Chafino, Marina Flores, Francesc Vidal, Hospital Universitari de Tarragona Joan XXIII (Tarragona); Marta Montero-Alonso, María Tasias-Pitarch, Eva Calabuig-Muñoz, Miguel Salavert-Lletí, Juan Fernández-Navarro, Rosa Blanes-Hernández, Jennifer Sánchez-Guevara, Hospital Universitario y Politécnico de La Fe (Valencia); Juan González-García, José Ignacio Bernardino, Ana Delgado-Hierro, José Ramón Arribas, Víctor Arribas, Juan Miguel Castro, Luis Escosa, Iker Falces, Pedro Herranz-Pinto, Alicia González-Baeza, María Luz Martín-Carbonero, Rafael Micán, Rocío Montejano, María Luisa Montes, Luis Ramos-Ruperto, Berta Rodés Soldevila, Talia Sainz, Elena Sendagorta, Carmen Busca, Joanna Cano-Smith, Rosa de Miguel, María del Mar Arcos-Rueda, Alejandro de Gea-Grela, Nerea Iniesta-Arandia, Alejandro Díez-Vidal, María Jesús Roldán-Cabrales, Carlos Oñoro-López, Jara Llenas García, Laura Lucía Checa Daimiel, Hospital Universitario La Paz/IdiPAZ (Madrid); José Ramón Blanco, Laura Pérez-Martínez, Valvanera Ibarra, Luis Metola, Mercedes Sanz, Estrella Melús, Hospital San Pedro Centro de Investigación Biomédica de La Rioja (CIBIR) (Logroño); Rosa Martínez-Álvarez, Desiré Gil-Pérez, Valentina Novarese, Ruth Caballero-Asensio, María Aranzazu Caudevilla-Martínez, María Espuelas-Monge, Hospital Universitario Miguel Servet (Zaragoza); David Dalmau, Marina Martinez, Angels Jaén, Mireia Cairó, Javier Martinez-Lacasa, Roser Font, Laura Gisbert, Hospital Universitari Mutua Terrassa (Terrassa); María Rivero, Carlos Ibero-Esparza, Estela Moreno García, Fernando Baigorria Feltrin, Ana Miqueleiz Zapatero, Patricia Moreira Escriche, Andrés Enrique Blanco di Matteo, Victoria Duro Suárez, Hospital Universitario de Navarra (Pamplona); Gemma Navarro, Sonia Calzado Isbert, Marta Navarro Vilasaro, Juan Francisco Delgado, Germa Julia Agulló, Parc Taulí Hospital Universitari (Sabadell); Ignacio de los Santos, Alejandro de los Santos, Lucio García-Fraile, Enrique Martín-Gayo, Ildefonso Sánchez-Cerrillo, Ángela Gutiérrez, Carmen Sáez, Ana Barrios Blandino, Azucena Bautista, Marianela Ciudad, María Aguilera García, Violeta Sampériz Rubio, Javier Pérez Serrano, Isabel Belmonte Martín de Santa Olalla, Hospital Universitario de La Princesa (Madrid); Santiago Moreno, Santos del Campo, José Luis Casado, Fernando Dronda, Ana Moreno, María Jesús Pérez-Elías, Sergio Serrano-Villar, María Jesús Vivancos-Gallego, Javier Martínez-Sanz, Alejandro Vallejo Tiller, Matilde Sánchez-Conde, José Antonio Pérez-Molina, José Manuel Hermida, Erick De La Torre Tarazona, Elena Moreno del Olmo, Laura Martín Pedraza, Claudio Díaz García, Jorge Díaz Álvarez, Alejandro García-García, Raquel Ron-González, Sergio Calderón Vicente, Roser Navarro Soler, Sara Saiz-Baggetto, Ana del Amo-de Palacios, Laura Luna, Miguel Antón Ámez Segovia, Sara Martín Colmenarejo, Cristina Chica, Hospital Universitario Ramón y Cajal (Madrid); Enrique Bernal, María Dolores Hernández, Antonia Alcaraz, Joaquín Bravo, Ángeles Muñoz Pérez, Cristina Tomás Jimenez, Salvador Valero Cifuentes, Eva García-Villalba, Román González Hipólito, Elena Guijarro-Westermeyer, Rodrigo Martínez-Rodríguez, José Miguel Gómez Verdú, Hospital General Universitario Reina Sofía (Murcia); Federico García, Clara Martínez, Maite Laperal, Leopoldo Muñoz Medina, Marta Álvarez-Estevez, Natalia Chueca-Porcuna, David Vinuesa-García, Adolfo de Salazar-González, Ana Fuentes-López, Emilio Guirao, Andrés Ruiz-Sancho, Francisco Anguita, Naya Faro, Lucia Chaves, Marta Illescas, Paloma Muñoz-Baez, Lucía Pérez, Ana Alberola Romano, Alberto Vazquez Blanquiño, Lucía Guillén-Zafra, Javier Martínez de Victoria-Carazo, Hospital Universitario Clínico San Cecilio (Granada); Jorge Del Romero-Guerrero, Montserrat Raposo, Teresa Puerta-López, Mar Vera, Juan Ballesteros, Begoña Baza, Laura Dans Villán, Ruben Linares Navarro, Ines Armenteros Yeguas, Eva Orviz-García, Santiago Fernández Castelao, Centro Sanitario Sandoval (Madrid); Antonio Antela, Sandra Daponte, Elena Losada, Hospital Clínico Universitario de Santiago (Santiago de Compostela); Melchor Riera, Antonio Vanrell, María Peñaranda, Mª Angels Ribas, Antoni A. Campins, Mercedes Garcia-Gasalla, Francisco J Fanjul, Javier Murillas-Angoiti, Luisa Martin-Pena, Francisca Artigues, Sophia Pinecki, Adrián Ferre, Hospital Universitario Son Espases (Palma de Mallorca); Jesús Santos, María López-Jódar, Cristina Gómez-Ayerbe, Isabel Viciana, Rosario Palacios, Hospital Universitario Virgen de la Victoria (Málaga); Luis Fernando López-Cortés, Silvia Llaves-Flores, Inmaculada Rivas Jeremías, Nuria Espinosa, Cristina Roca-Oporto, Marta Herreros-Romero, César Sotomayor de la Piedra, Abraham Saborido Alconchel, Manuel Francisco Liroa, Jesús Fernández Plaza; Hospital Universitario Virgen del Rocío (Sevilla); Juan Manuel Tiraboschi, Camila Soledad Piatti, Arkaitz Imaz, María Saumoy, Analuz Fernandez, Jaime Vega Costa, Daniel Medina Gamito, Hospital Universitario de Bellvitge (Hospitalet de Llobregat); Adrián Curran, Vicenç Falcó, Jordi Navarro, Joaquin Burgos, Paula Suanzes, Jorge García, Vicente Descalzo, Patricia Álvarez, Bibiana Planas, Marta Sanchíz, Arnau Monforte, Hospital Universitario Valle de Hebrón (Barcelona); Julián Olalla, Javier Pérez, Alfonso del Arco, Javier de la Torre, Francisca Ruiz, Hospital Costa del Sol (Marbella); Onofre Juan Martínez, Lorena Martinez, Francisco Jesús Vera, Josefina García, Begoña Alcaraz, Sergio Guillén Martínez, Patricia Carles García, Hospital General Universitario Santa Lucía (Cartagena); Álvaro Mena de Cea, Brais Castelo, Pilar Vázquez-Rodríguez, Soledad López-Calvo, Verónica Pazos López, Complejo Hospitalario Universitario a Coruña (CHUAC) (A Coruña); Sofía Ibarra-Ugarte, Guillermo García, Josu Mirena, Oscar Luis Ferrero, Mireia de la Peña, Miriam López, Iñigo López-Azkarreta, Itxaso Lombide-Aguirre, Víctor Polo, Joana de Miguel, Beatriz Ruiz-Estevez, Maite Ganchegui-Aguirre, María Jesús Barberá-Gracia, Mónica Vélez Tobarias, Isabel Izuzquiza-Avanzini, Jimena Varona Pérez, María Iriarte-Ibarrarán, Hospital Universitario Basurto (Bilbao); Carlos Galera, Marian Fernández, Helena Albendin, Antonia Castillo, Asunción Iborra, Antonio Moreno, María Angustias Merlos, Almudena Ortuño, Hospital Universitario Virgen de la Arrixaca (El Palmar); Concha Amador, Francisco Pasquau, Concepción Gil, José Tomás Algado, Hospital de la Marina Baixa (La Vila Joiosa); Inés Suarez-García, Eduardo Malmierca, Patricia González-Ruano, Mª Pilar Ruiz, Luz Balsalobre, Ángela Somodevilla, Rebeca Fuerte Martínez, Hospital Universitario Infanta Sofía (San Sebastián de los Reyes); María de la Villa López, Mohamed Omar, Carmen Herrero, Hospital Universitario de Jaén (Jaén); Miguel Alberto de Zárraga, Desirée Pérez, Carlos Tarrazo, Juan Valdes Becares, Hospital Universitario San Agustín (Avilés); Vicente Estrada, Nieves Sanz, Noemí Cabello-Clotet, María José Núñez, Ana Muñoz, Juncal Pérez-Somarriba, Reynaldo Homen, Rafael Rubio-Martín, Susana Olmedo, Julia Barrado; Hospital Clínico San Carlos (Madrid); Miguel Górgolas, Alfonso Cabello, Cristina Algar Arévalo, Beatriz Álvarez, Laura Prieto, Aws Al-Hayani, Irene Carrillo, Marta López de las Heras, Hospital Universitario Fundación Jiménez Díaz (Madrid); José Sanz, Cristina Hernández-Gutiérrez, María Novella-Mena, Hospital Universitario Príncipe de Asturias (Alcalá de Henares); María José Galindo, Sandra Pérez Gómez, Ana Ferrer, Anaïs Corma Gómez, Hospital Clínico Universitario de Valencia (Valencia); Antonio Rivero-Román, Laura Ruiz-Torres, Antonio Rivero-Juárez, Pedro López-López, Mario Frias-Casas, Ángela Camacho, Ignacio Pérez, Diana Corona, Javier Manuel Caballero, Marina Gallo Marín, María Casares, Lucía Ríos-Muñoz, Claudia Ferreira-Tata, María Carrasquilla, Hospital Reina Sofía (Córdoba); Rafael Rodríguez-Rosado Martinez-Echevarría, Hospital Universitario Severo Ochoa (Leganés); Juan Macías Sánchez, Pilar Rincón, Luis Miguel Real, Jésica Martín-Carmona, Hospital Universitario Virgen de Valme (Sevilla); Eva Poveda, Alexandre Pérez, Luis Morano, Celia Miralles, Antonio Ocampo, Jacobo Alonso, Inés Martínez, Aida López-López, Hospital Álvaro Cunqueiro (Vigo); Carlos Dueñas, Pablo Telleria, Laura Rodríguez-Fernández, Marta de la Fuente López, Marina García de Vicuña, Sara Gómez García, Alberto Rodríguez-Iglesias, Hospital Clínico Universitario de Valladolid (Valladolid); Roberto Güerri Fernández, Angela Requena Company, Juan Du, Agustin Marcos Blanco, Itziar Arrieta Aldea, Esperanza Cañas-Ruano, Cecilia Canepa, Natalia García Giralt, Jade Soldado, Irene Carbonell, María José Fernández Quiroga, Hospital del Mar (Barcelona); Carlos de Andrés David, Karenina Antelo Cuéllar, Inmaculada Poquet Catalá, María Josefa de la Asunción Villaverde, Laira Navarro Peiró, Hospital de Dénia (Dénia); Jorge Sánchez Villegas, Virginia Palomo Jiménez, Aurora Alemán Rodríguez, Lola Cubero Aranda, Hospital Universitario Virgen de la Macarena (Sevilla); M Carmen Fariñas Álvarez, Claudia González Rico, Noelia Ruiz Alonso, Carlos Armiñanzas Castillo, Francisco Arnaiz de las Revillas Almajano, Manuel Gutiérrez Cuadra, Raúl Parra Fariñas, Paula Runza Buznego, Aitziber Illaro Uranga, Hospital Universitario Marqués de Valdecilla (Santander); José Luis Blanco Arévalo, Pilar Callau Cabrera, Josep Mallolas Masferrer, José Alcamí Pertejo, Sonsoles Sánchez-Palomino, Núria Climent, Ana González-Cordón, Montse Laguno Centeno, María Martínez-Rebollar, Juan Ambrosioni, Berta Torres, Lorena de la Mora, Alexy Inciarte, Esteban Martínez, José María Miró, Abiu Sempere, Julia Calvo, Iván Chivite, David García, Alberto Foncillas, Daniela Malano, Montse Plana, Roger Llobet, Estela Solbes, Octavi Roman, Rona Sagarra, Vanesa Guilera, Gemma Olmeda, Paula Arreba, María José Rodríguez, Emma Fernández, Ana Rodríguez, Alba ortega, Sergi Anguera, Raquel Aguiló, Laura Novell, Hospital Clínic de Barcelona (Barcelona); Luis Miguel Bedoya Del Olmo, Manuela Beltran Vicente, Mercedes Bermejo Herrero, Maria Esther Calonge Errejon, Laura Capa Muñoz, Maria Teresa Coiras Lopez, Francisco Diez Fuertes, Javier Garcia Perez, Nuria Gonzalez Fernandez, Elena Mateos De Las Moreras, Maria Teresa Perez Olmeda, Victor Sanchez Merino, Maria Eloisa Yuste Herranz, Centro Nacional de Microbiología (Majadahonda); Carmen Elena Gomez Rodriguez, Juan Francisco Garcia Arriaza, Laura Sin Diaz, Beatriz Perdiguero de la Torre, Laura Marcos Villar, Patricia Perez Ramirez, Enrique Alvarez Coruña, David Astorgano Lopez, Cristina Sanchez Corzo, Centro Nacional de Biotecnología. Consejo Superior de Investigaciones Científicas (CSIC) (Madrid).
