# Supplementary material for: Validity evidence for the Clinic Screening Tool for HIV (CST-HIV) addressing factors that undermine health-related quality of life in people living with HIV
Source: Front Psychol. 2026 Oct 16;17:1940441. doi: 10.3389/fpsyg.2026.1940441 (PMC13719464; doi:10.3389/fpsyg.2026.1940441)
Supplement: Supplementary file 1 [file Data_Sheet_1.PDF]

Supplementary Information

Validity evidence for the brief screening tool (CST-HIV) addressing factors that undermine health-related quality of life in people with HIV

María José Fuster-RuizdeApodaca[1,2], Patricia Recio[1], Trenton M. White[3,4], Carlos Iniesta[2,5], Rebeca Izquierdo[5,6], Julián Alexander Portocarrero[2], Jeffrey V. Lazarus[3,4], Inmaculada Jarrín[5,6]; CoRIS cohort[†]

1. Faculty of Psychology, Universidad Nacional de Educación a Distancia (UNED), Madrid, Spain
2. Spanish Interdisciplinary AIDS Society (SEISIDA), Madrid, Spain
3. Barcelona Institute for Global Health (ISGlobal), Barcelona, Spain
4. City University of New York School of Public Health and Health Policy (CUNYSPH), New York, New York, USA
5. CIBERINFEC, Carlos III Health Institute, Madrid, Spain
6. CoRIS Cohort Coordination Unit, National Centre for Epidemiology, Carlos III Health Institute, Madrid, Spain.

[†]Group authorship: Centers and investigators participating in CoRIS cohort are listed in the Acknowledgments.

Corresponding author: Trenton M. White, PhD, Trenton.white@isglobal.org, Carrer de Rosselló 171, Entresuelo 1

CST-HIV Questionnaire

Dimension	Nº	Item in Spanish	Item in English
Anticipated stigma	1	En el último mes, ¿hasta qué punto te ha preocupado contarle a alguien que tienes VIH?	During the past month, to what extent have you been worried about telling someone you have HIV?

	2	En el último mes, ¿hasta qué punto te ha preocupado que la gente te juzgue si se enteran que tienes VIH?	During the past month, to what extent have you been worried about people judging you if they learn you have HIV?
	3	En el último mes, ¿hasta qué punto te ha preocupado pensar que la gente te va a rechazar por tener VIH?	During the past month, to what extent have you been worried about people rejecting you for having HIV?
Emotional distress	4	En el último mes, ¿con que frecuencia has sentido ansiedad?	During the past month, how often have you felt anxiety?
	5	En el último mes, ¿con que frecuencia has tenido sentimientos de tristeza o decaimiento?	During the past month, how often have you felt sadness or felt discouraged?
	6	En el último mes, ¿con que frecuencia has sentido temor al futuro?	During the past month, how often have you felt fearful of the future?
Sexuality	7	En el último mes, ¿en qué medida te has sentido satisfecho/a con tu vida sexual?	During the past month, how satisfied have you felt with your sex life?
	8	En el último mes, ¿en qué medida ha disminuido tu deseo sexual o tu interés en tener sexo?	During the past month, has your sex drive or your interest in sex decreased?
	9	En el último mes, ¿en qué medida ha afectado el VIH negativamente a tu vida sexual?	During the past month, has HIV negatively affected your sex life?
Social support	10	En el último mes, ¿con que frecuencia has tenido personas a tu alrededor en las que apoyarte en caso de necesitarlo?	During the past month, how often have you had people around you whom you can lean on in case of need?
	11	En el último mes, ¿con que frecuencia has tenido alguien de confianza para hablar de tus problemas?	During the past month, how often have you had someone you trust to speak to about your problems?

	12	En el último mes, ¿con que frecuencia has tenido gente que te haya hecho sentirte querido/a?	During the past month, how often have people made you feel loved?
Material deprivation	13	En el último mes, ¿hasta qué punto has tenido suficiente dinero para cubrir tus necesidades?	During the past month, have you had enough money to meet your needs?
	14	En el último mes, ¿hasta qué punto has estado satisfecho/a con la calidad del lugar donde vives?	During the past month, how satisfied have you been with the quality of the place where you live?
	15	En el último mes, ¿hasta qué punto has tenido dinero para realizar actividades de ocio?	During the past month, have you had money for leisure activities?
Sleep and fatigue	16	En el último mes, ¿hasta qué punto has tenido problemas de sueño?	During the past month, have you had sleep problems?
	17	En el último mes, ¿hasta qué punto te has sentido satisfecho/a con la calidad de tu sueño?	During the past month, how satisfied have you felt with the quality of your sleep?
	18	En el último mes, ¿hasta qué punto te has sentido cansado/a?	During the past month, how tired have you felt?
Cognitive problems	19	En el último mes, ¿hasta qué punto has tenido dificultad para pensar con claridad?	During the past month, have you had difficulty thinking clearly?
	20	En el último mes, ¿hasta qué punto has tenido dificultad para prestar atención?	During the past month, have you had difficulty paying attention?
	21	En el último mes, ¿hasta qué punto crees que te ha costado más aprender cosas nuevas?	During the past month, do you think that it has been harder for you to learn new things?

Physical symptoms	22	En el último mes, ¿hasta qué punto has tenido cambios molestos en tu apariencia corporal, como acúmulos de grasa, ganancia o pérdida de peso?	During the past month, have you experienced unpleasant body changes such as fat accumulation, weight gain, or weight loss?
	23	En el último mes, ¿hasta qué punto has tenido dolor en alguna parte del cuerpo? (por ejemplo, dolor de cabeza, articulaciones, pinchazos en los músculos, etc.)	During the past month, have you felt pain somewhere in your body? (for example, headache, joint pain, muscle cramps)
	24	En el último mes, ¿hasta qué punto has tenido molestias digestivas (dolor de estómago, gases, diarrea, nauseas o vómitos)	During the past month, have you suffered digestive problems? (stomach pain, flatulence, diarrhea, nausea, or vomiting)

Supplementary Results

CST-HIV Scores

The distribution of scores across the eight CST-HIV domains is shown in Supplementary Table 2 Anticipated stigma and psychological distress, while present to a moderate extent in the sample, did not reach particularly high levels for most people with HIV. Sexuality and social support domains showed intermediate mean scores, suggesting that both sexual concerns and perceived support are relevant but not uniformly problematic across the cohort. Scores relating to material deprivation, sleep and fatigue, cognitive complaints, and physical symptoms also occupied the middle portion of their respective scales, indicating diverse but generally moderate burdens in these areas.

Construct validity: Confirmatory Factor Analysis

The eight hypothesised dimensions of the CST-HIV had an overall Kaiser-Meyer-Olkin (KMO) measure of 0.813, and Bartlett's test of sphericity was significant ($\chi^2 = 4471.78$, df = 276, $p < .001$), confirming the adequacy of factor analysis.

The eight-factor model also demonstrated a good fit to the data with a chi-square to degrees of freedom ratio (χ^2/df) of 1.79, although the chi-square test was significant ($\chi^2 = 400.61$, df = 224, $p < .001$), as expected given the sample size. Fit indices supported the adequacy of the model: CFI = 0.972, TLI = 0.965, NNFI = 0.965, NFI = 0.939, IFI = 0.972, RNI = 0.972, and GFI = 0.993. The RMSEA was 0.053 (90% CI: 0.044–0.061; $p = .296$), and the SRMR was 0.054. Standardised factor loadings for individual items were all significant ($p < .001$) and ranged from moderate to high across the eight dimensions—most exceeding traditional thresholds for strong item-factor association (e.g., all psychological distress items > 0.76; cognitive complaints > 0.81, etc.). The Average Variance Extracted (AVE) for each domain was acceptable, with most factors above 0.5, indicating good convergent validity, and all factor reliabilities (coefficient ω and α) were within acceptable ranges (e.g., $\omega = 0.89$ for anticipated stigma, 0.86 for psychological distress, and 0.88 for social support) (Supplementary Table 1).

All factor covariances were positive and statistically significant, ranging from low to moderate in strength, and the heterotrait-monotrait ratios supported sufficient discriminant validity between factors.

Evidence of Convergent and Discriminant Validity

To examine convergent validity, the eight dimensions of the CST-HIV were correlated with a set of widely used external instruments with well-established psychometric properties that assess constructs theoretically related to the specific burden domains targeted by each CST-HIV dimension (see Supplementary Table 3).

The pattern of associations supported the convergent validity of the CST-HIV dimensions. Specifically, Anticipated Stigma showed a strong positive correlation with the Spanish Stigma Scale ($r = .65$, $p < .001$), together with small-to-moderate negative correlations with disease-specific (WHOQOL-HIV-BREF; $r = -.28$, $p < .001$) and generic (EQ-5D-5L; $r = .16$, $p < .05$) quality of life indicators. Emotional Distress was very strongly associated with psychological distress measured by the PHQ-4 ($r = .79$, $p < .001$) and showed substantial correlations with sleep problems (ISI; $r = .55$, $p < .001$), fatigue severity (FSS; $r = .59$, $p < .001$), and poorer quality of life (WHOQOL-HIV-BREF; $r = -.71$, $p < .001$). The Sexuality dimension correlated strongly and inversely with satisfaction with sex life assessed by PROMIS ($r = -.74$, $p < .001$), indicating that greater perceived sexual burden was associated with lower sexual satisfaction. Social Support exhibited strong negative correlations with both confidant support ($r = -.69$, $p < .001$) and affective support ($r = -.67$, $p < .001$), and a positive association with social exclusion (SEI-HS; $r = .42$, $p < .001$). In addition, Material Deprivation was strongly related to social exclusion ($r = .67$, $p < .001$) and moderately to strongly associated with poorer quality of life (WHOQOL-HIV-BREF; $r = -.57$, $p < .001$). The Sleep and Fatigue dimension showed very strong correlations with insomnia severity (ISI; $r = .79$, $p < .001$) and fatigue severity (FSS; $r = .44$, $p < .001$), while Cognitive Complaints was substantially associated with perceived cognitive functioning as measured by NeuroQoL ($r = -.64$, $p < .001$). Finally, Physical Symptoms demonstrated moderate-to-strong associations with sleep problems (ISI; $r = .44$, $p < .001$), fatigue (FSS; $r = .44$, $p < .001$), and reduced quality of life (WHOQOL-HIV-BREF; $r = -.51$, $p < .001$). Overall, correlations were consistently stronger with conceptually proximal measures than with theoretically distinct constructs, supporting both the convergent and discriminant validity of the CST-HIV.

Criterion validity

We found a significant negative correlation between $CD4^+$ cell/mm^3 count and the CST-HIV dimension *Anticipated Stigma* (Spearman's rho = -.16; $p = .014$; N =231). Moreover, the dimension *Material Deprivation* correlated negatively with $CD4^+$ cell count (Spearman's rho = -.18; $p = .005$), with $CD4^+$ cell percentage (Spearman's rho = -.14; $p = .040$), and showed a small, non-significant positive correlation with viral load when analyzed on the log10 scale at Tc (e.g., $\rho \approx .12$; $p \approx .06$).

Sleep and fatigue showed a small, positive correlation with viral load when using raw copies/mL (r = 0.159, p = 0.016; N=226), while the association was not significant on the log10 scale (r = 0.116, p = 0.083).

Regarding $CD4^+$ cell count, GLM results did not show significant differences in between age groups at either time point. Multivariate tests showed no significant main effect for Time ($CD4^+$ cell count, Wilks' $\lambda = .996$, $F(1, 167) = 0.66$, $p = .419$, $\eta^2 = .004$), nor for the interaction between Time and any CST dimensions or Age group. None of the interactions between Time and CST dimensions reached statistical significance (all $p > .05$), indicating that changes in $CD4^+$ cell counts across time points were not significantly associated with

the individual CST domains or with age group. Regarding between-subjects effects, we found that of the CST dimensions, only *Psychological distress* was statistically significantly associated with overall $CD4^+$ cell count across time points ($F(1, 167) = 3.97$, $p = .048$, partial $\eta^2 = .023$). Consistent with this effect, participants with higher *Psychological Distress* scores showed higher retrospective $CD4^+$ cell counts ($B = 27.82$, $SE = 12.42$, $t = 2.24$, $p = .026$). None of the other CST domains or dichotomised age groups were significantly associated with $CD4^+$ cell count (all $p > .05$).

The repeated measures model indicated no statistically significant associations or effects for $CD4^+$ cell percentage, either for main effects or interactions (all $p > .05$).

For viral load analyzed on the log10 scale, the overall change over time was significant (p =.0.29), indicating a small reduction from the retrospective to the cross-sectional time point. Among the CST dimensions, only Anticipated Stigma showed a significant interaction with Time ($p = .023$) and a small but significant between-subjects association with mean viral load ($\beta \approx +0.11$ log10 per 1 SD; $p = .042$), indicating that participants with higher anticipated stigma had higher viral loads at the retrospective time point, with attenuation of this difference at the cross-sectional assessment, independently of age and other CST domains.

Comparisons across well-known groups

Group comparisons based on age categories—participants younger than 50 years (n = 198) versus those aged 50 or older (n = 88)—revealed a statistically significant difference only in the domain of *Sexual Satisfaction*. Participants under 50 years reported higher mean scores (M = 7.71, SD = 2.86, SE = 0.20) compared to those aged 50 or older (M = 6.51, SD = 3.04, SE = 0.32). The independent-samples t-test confirmed that this difference was significant (t = -3.664, df = 284, $p < .001$), with a moderate effect size (Cohen's $d = -0.47$, SE = 0.13). For all other CST-HIV dimensions no significant age-related differences were observed.

No statistically significant differences were observed in the CST-HIV dimensions according to AIDS diagnosis background nor gender.

When comparing self-reported outcomes by sexual orientation (heterosexual vs. MSM), a statistically significant difference emerged only in the domain of *Physical Symptoms*. Heterosexual participants reported a higher burden of physical symptoms compared to MSM, with an independent samples t-test indicating a significant difference ($t = -2.20$, $df = 272$, $p = .028$), corresponding to a small-to-moderate effect size (Cohen's $d = -0.39$, SE = 0.18).

Supplementary Tables

Supplementary Table 1. Health-related data

Health-related variable	Response category	N (%)
AIDS history,Tc	Yes	3 (1%)
AIDS history,Tr	Yes	33 (11.5%)
Weekly alcohol consumption , Tc (Units, M±SD)		1.9±4.6
Weekly alcohol consumption , Tr (Units, M±SD)		2.4±5.9
Injection drug use, Tc	Yes	1 (0.3%)
Injection drug use, Tr	Yes	0 (0%)
Smoker, Tc	Yes	43 (15%)
Cigarettes per day_Tc (M±SD)		10.9±8.6
Smoker, Tr		72 (25.1%)
Cigarettes per day_Tr (M±SD)		12.7±9.9
CD4 cells/mm^3_Tc (M±SD)		780.3±371.3
CD4 %_Tc (M±SD)		33.2±10.5
CD4 cells/mm^3_Tr (M±SD)		812.1±338.5
CD4 %_Tr (M±SD)		32.8±9.4
AIDS events between Tr and Tc	Yes	3 (1%)
AIDS events before Tr	Yes	33 (11.5%)
Non-AIDS events between Tr and Tc	Yes	12 (4.2%)
Number of non-AIDS events between Tr and Tc (M±SD)		0.04±0.20
Non-AIDS events before Tr	Yes	48 (16.8%)
Number of non-AIDS events before Tr (M±SD)		0.23±0.61
Polypharmacy, Tc	Yes	41 (14.3%)
Polypharmacy, Tr	Yes	125 (43,7%)
Number of medicines_Tc (M±SD)		0.20±0.54
Number of medicines, Tr (M±SD)		0.91±1.4

Note: M = Mean; SD = Standard Deviation; Tc = Time crossectional, Tr = Time retrospective

Supplementary Table 2. Descriptive Statistics, Reliability and Convergent Validity of the CST-HIV Scale Factors

	**Mean (SD)**	**ω**	**α**	**AVE**
F1. Anticipated Stigma	9.85 (3.75)	0.911	0.862	0.755
F2. Psychological Distress	7.76 (3.01)	0.890	0.849	0.725
F3. Sexuality	8.13 (2.99)	0.736	0.723	0.500
F4. Social Support	6.44 (3.35)	0.882	0.878	0.737
F5. Material Deprivation	6.77 (2.52)	0.703	0.768	0.557
F6. Sleep and Fatigue	8.91 (2.84)	0.799	0.800	0.593
F7. Cognitive Complaints	6.59 (2.93)	0.918	0.882	0.784
F8. Physical symptoms	7.27 (2.85)	0.741	0.693	0.474

Note. SD = Standard Deviation; AVE = Average Variance Extracted; ω = McDonald's Omega; α = Cronbach's Alpha.

Supplementary Table 3. Pearson Correlation Matrix among Study Variables

Variable	1	2	3	4	5	6	7	8	9	10	11	12	13	14	15	16	17	18	19
1. Anticipated Stigma CST	--	0.27***	0.29***	0.21***	0.10	0.15*	0.17**	0.11	0.65***	0.19**	-0.15*	-0.27***	-0.25***	0.18**	0.20***	0.08	-0.08	-0.28***	0.16*
2. Emotional Distress CST		--	0.38***	0.39***	0.34***	0.62***	0.58***	0.45***	0.22***	0.79***	-0.27***	-0.43***	-0.45***	0.55***	0.59***	0.38***	-0.41***	-0.71***	0.58***
3. Sexuality CST			--	0.32***	0.17**	0.30***	0.27***	0.19**	0.21***	0.37***	-0.74***	-0.30***	-0.29***	0.23***	0.27***	0.15*	-0.14*	-0.46***	0.34***
4. Social Support CST				--	0.37***	0.27***	0.23***	0.23***	0.23***	0.34***	-0.30***	-0.69***	-0.67***	0.60***	0.24***	0.17**	-0.05	-0.49***	0.29***
5. Material Deprivation CST					--	0.35***	0.36***	0.38***	0.12*	0.39***	-0.18**	-0.42***	-0.43***	0.67***	0.30***	0.27***	-0.28***	-0.57***	0.33***
6. Sleep and Fatigue CST						--	0.49***	0.45***	0.12*	0.61***	-0.21***	-0.32***	-0.32***	0.42***	0.79***	0.44***	-0.40***	-0.61***	0.54***
7. Cognitive Complaints CST							--	0.46***	0.12*	0.61***	-0.17**	-0.27***	-0.30***	0.43***	0.52***	0.43***	-0.64***	-0.62***	0.47***
8. Physical Symptoms CST								--	0.13*	0.52***	-0.15*	-0.26***	-0.26***	0.44***	0.44***	0.44***	-0.37***	-0.51***	0.49***
9. Spanish Stigma Scale									--	0.20***	-0.07	-0.23***	-0.23***	0.22***	0.21***	0.11	-0.11	-0.27***	0.18**
10. PHQ-4 distress										--	-0.25***	-0.43***	-0.44***	0.59***	0.60***	0.47***	-0.51***	-0.66***	0.64***
11. PROMIS Sex Life 2											--	0.31***	0.30***	-0.25***	-0.19**	-0.10	0.11	0.43***	-0.29***
12. Confidant Support												--	0.87***	-0.66***	-0.32***	-0.14*	0.22***	0.60***	-0.39***
13. Affective Support													--	-0.69***	-0.30***	-0.16**	0.22***	0.61***	-0.40***
14. Social Exclusion														--	0.42***	0.30***	-0.31***	-0.67***	0.45***
15. Sleep problems															--	0.49***	-0.48***	-0.63***	0.55***
16. Fatigue																--	-0.42***	-0.63***	0.46***
17. Neurocognitive																	--	-0.45***	-0.44***
18. WHOQOL quality of life																		--	-0.67***
19. EQ-5D-5L quality of life																			--

Note. *p < .05, **p < .01, ***p < .001. Spanish Stigma Scale = factors of disclosure concerns and public stigma of the Spanish Stigma Scale, PHQ-4 distress = Patient Health Questionnaire-4, PROMIS Sex Life 2 = PROMIS V2.0 Satisfaction with Sex Life scale, Confidant Support = Confidant Support factor of Duke-UNC Functional Social Support Questionnaire, Affective Support = Affective Support factor of Duke-UNC Functional Social Support Questionnaire, Social Exclusion = Spanish adaption version of the Social Exclusion Index for Heath Surveys (SEI_HS), Sleep Problems = Spanish version of the Insomnia Severity Index (ISI), Fatigue = Spanish version of the Insomnia Severity Index (ISI), Neurocognitive = NeuroQol Cognitive Function Scale, WHOQOL quality of life = WHOQOL-HIV-BREF.
